# The Essential Oil from *Oliveria decumbens* Vent. (Apiaceae) as Inhibitor of Breast Cancer Cell (MCF-7) Growth

**DOI:** 10.3390/ph16010059

**Published:** 2022-12-30

**Authors:** Mandana Shariatzadeh, Akbar Karami, Ali Moghadam, Mahbobeh Lotfi, Filippo Maggi, Esmaeil Ebrahimie

**Affiliations:** 1 Institute of Biotechnology, Shiraz University, Shiraz 71441, Iran; 2 Department of Horticulture Science, School of Agriculture, Shiraz University, Shiraz 71441, Iran; 3 Chemistry Interdisciplinary Project (ChIP) Research Center, School of Pharmacy, University of Camerino, 62032 Camerino, Italy; 4 Genomics Research Platform, School of Agriculture, Biomedicine and Environment, La Trobe University, Melbourne, VIC 3000, Australia; 5 School of Animal and Veterinary Sciences, The University of Adelaide, Adelaide, SA 5371, Australia; 6 School of BioSciences, The University of Melbourne, Melbourne, VIC 3010, Australia

**Keywords:** breast cancer, PTEN protein, Aurora kinase A, essential oil, apoptosis, *Oliveria decumbens*

## Abstract

*Oliveria decumbens* Vent. is an aromatic and medicinal plant traditionally used in Iran for the treatment of infections, gastrointestinal diseases, cancer, and inflammation. This research was aimed at investigating the pharmacological potential of *O. decumbens* essential oil (OEO) and its main compounds, focusing on OEO’s cytotoxic effects on MCF-7 breast cancer cells. OEO was obtained by hydro-distillation, and the chemical constituents were identified using GC-MS. Thymol, carvacrol, γ-terpinene, and *p*-cymene were the main OEO constituents. When MCF-7 cells were treated with OEO, the expressions of genes related to apoptosis (*BIM* and *Bcl-2*), tumor suppression (*PTEN*), and cell growth inhibition (*AURKA*), were evaluated using real-time PCR. Moreover, molecular docking was used for studying in silico the interaction of OEO principal compounds with PTEN and AURKA. The expression of *AURKA* was significantly reduced since the OEO treatment enhanced the expression of *PTEN*. Through in silico molecular docking, it was revealed that thymol, carvacrol, *p*-cymene, and γ-terpinene can activate PTEN and thus inhibit AURKA. Additionally, the DNA fragmentation assay, acridine orange/ethidium bromide (AO/EB) double-staining assay, and real-time PCR highlighted the fact that the OEO treatment could activate apoptosis and inhibit cell proliferation. Therefore, OEO is a viable candidate to be employed in the pharmaceutical industry, specifically as a possible agent for cancer therapy.

## 1. Introduction

Genetic damage in cells that exhibit defects in division and mutation is the main cause of cancer. As a serious threat to personal health, breast cancer is the cause of many deaths among women annually and worldwide [1,2]. Cell proliferation, angiogenesis, and death (apoptosis) are regulated by PTEN, a critical signaling molecule that plays a main role in several physiological processes [3]. PTEN is a lipid phosphatase of PI 3-kinase that works to prevent phosphatidylinositol-3 kinase (PI3K) signaling by converting the phosphatidylinositol (3,4,5)-triphoshphate (PIP3) into PIP2 [4]. By blocking the PI3K/AKT/mTOR pathway, PTEN plays a crucial role in regulating intracellular signaling for cell growth and proliferation [5]. The human *AURK* gene is a member of the serine/threonine kinase family. In human malignancies, Aurora kinases become over-expressed and amplified. Aurora kinase A (*AURKA*) is over-expressed in cancer and has various roles in carcinogenesis, e.g., the disruption of microtubule stability and cell cycle arrest by phosphorylating the RAS-association domain family 1 [6,7].

There are various types of plant-based anticancer drugs that act against proliferating cells [8]. Plants are recognized as a rich source of biologically active compounds that can be effective in the treatment of chronic diseases [9]. Compared to current breast cancer treatment methods, such as radiology and chemotherapy, plant-based compounds have fewer toxic side-effects. Essential oils (EOs) may be considered an appropriate alternative for cancer treatment [10]. In addition, there are several plant-originated anticancer and antimicrobial compounds, including secondary metabolites and proteins, that entail fewer side-effects [10,11]. Multiple lines of cancer cells are targeted by EOs and their major compounds [12]. EOs can have an antiproliferative impact through a variety of mechanisms, such as cell membrane rupture and apoptosis induction. EOs are considered complex mixtures of volatile compounds which include, to a major extent, terpenoids (mono- and sesquiterpenes) and phenylpropanoids. The chemical constituents of EOs vary among different species and subspecies [13]. Many plant-based EOs have demonstrated antiproliferative activity on MCF7 cells. EOs with potential as chemopreventive agents have been obtained from several plant species such as *Schefflera heptaphylla* (L.) Frodin [14], *Heteropyxis dehniae* Suess. [15], *Satureja khuzistanica* Jamzad [16], and *S. intermedia* C.A.Mey. [17].

*O. decumbens* Vent. is a herbaceous plant belonging to Apiaceae which is endemic to Iran and is found in southern and western regions of this country. The plant’s aerial parts are traditionally used in the Persian medicine for treating diarrhea, dyspepsia, abdominal pains, and fever [13,18]. Although the antimicrobial and antioxidant activities of *O. decumbens* essential oil (OEO) have already been studied, its anticancer activities remain to be clarified in detail [19,20,21].

The traditional background and medicinal effects reported in the literature inspired the current research on the anticancer potential of OEO. Here, the main compounds of OEO were examined for their impact on PTEN activation and AURKA inactivation. Indeed, their interactions with PTEN, a disruptive regulator of the PI3K/AKT signaling pathway, and AURKA have yet to be delineated. Thus, the interaction and effective binding between the main OEO compounds and protein targets of PTEN and AURKA have been studied for their cancer treating potential via in silico molecular docking. In addition, the expressions of genes involved in tumor suppression (*PTEN*), cell growth inhibition (*AURKA*), and apoptosis (*BIM* and *Bcl-2*) have been measured in cells exposed to OEO.

## 2. Results

### 2.1. Chemical Identification by GC/MS (Gas Chromatography–Mass Spectrometry) Analysis

The chemical compounds of OEO were identified by GC/MS. Thymol (26.63%), carvacrol (24.12%), γ-terpinene (19.6%), and *p*-cymene (19.95%) were the main constituents (Table 1).

### 2.2. Assessment of Antioxidant Activity

DPPH inhibitory activity is considered an important parameter for evaluating the antioxidant potential of chemical compounds of plant origin. The antioxidant activity of tert-butylhydroquinone (TBHQ) as positive control was also determined. The IC_50_ of OEO was 0.582 ± 0.011 mg/mL, whereas that of TBHQ was 0.030 ± 0.001 mg/mL.

### 2.3. Cytotoxic Activity

In vitro cytotoxic activities of OEO and doxorubicin (positive control) were expressed as IC_50_ values which indicated the required dose for 50% inhibition of cell growth (Table 2).

### 2.4. Alteration in the Morphology of MCF-7 Cells by Acridine Orange/Ethidium Bromide (AO/EB) Double-Staining

**Figure 1:**
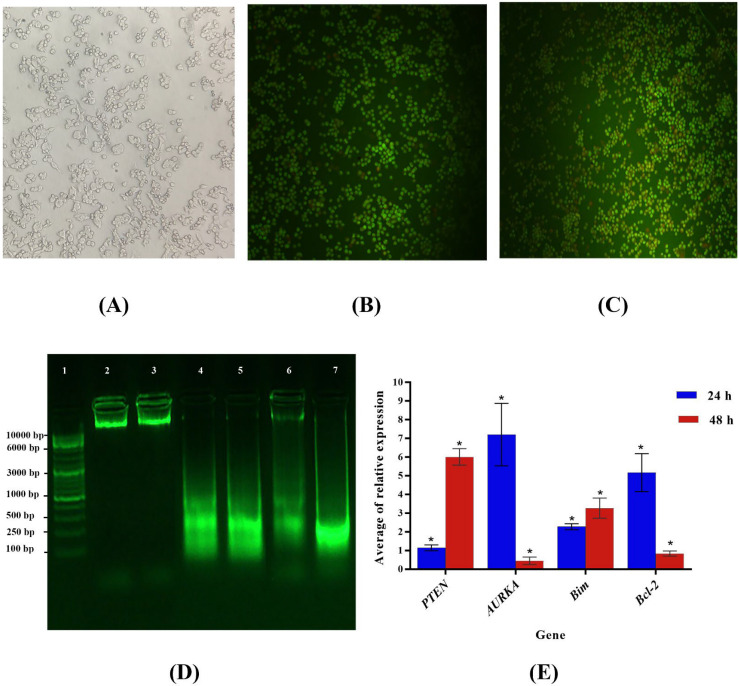
Treatment of MCF-7 cells with OEO essential oils for 24 h led to rapid morphological changes, including cellular contraction, irregular cell shapes, and cellular rounding under an inverted microscope (20×) (**A**). Typical morphological changes of MCF-7 cells induced by 39.54 μg/mL OEO, stained with AO/EB. The images were taken using fluorescence microscopy. The control group, i.e., no treated cells (**B**); OEO treated cells (**C**). Analysis of DNA from the control group (media culture), doxorubicin, and OEO-treated cells by agarose gel electrophoresis. Lane 1: DNA ladder; lane 2 and 3: DNA of the control group (media culture); lane 4 and 5: DNA from doxorubicin-treated cells; lane 6 and 7: DNA from OEO-treated cells (**D**), and the effect of OEO on expression levels of PTEN, AURKA, BIM, and Bcl-2 in the MCF-7 cell line. Data are presented as mean values ± SD, *n* = 3. (*) indicate significant differences caused by the essential oil (*p* < 0.01) (**E**).

Figure 1A shows that after treatment with OEO, significant morphological changes occurred in the cells. According to this assay, the color of the nucleus in healthy cells, original apoptotic cells, cells with secondary apoptosis, and necrotized cells was yellow, yellow to orange, dark orange, and red, respectively. Ethidium bromide was only detected in cells with damaged membranes. Although acridine orange penetrates into both dead and living cells, only the nucleus of the latter was green colored [22]. Therefore, the color of living cells was generally observed as green (Figure 1B,C). Apoptosis occurred in all treatments with OEO.

### 2.5. DNA Fragmentation Assay

Apoptosis is characterized by changes in morphology of cells and their nuclei. These changes may occur as cellular contraction, density, and fragmentation of nuclei and plasma membranes. In most cases, this process is associated with chromosomal DNA destruction. The DNA ladder pattern can also be observed in the apoptosis process [23]. After treating the cells with appropriate concentrations of OEO, DNA was extracted using the CTAB method. Figure 1D shows that the DNA ladder was observed in the assayed cultures after treatment with doxorubicin and OEO.

### 2.6. Expression of BIM, Bcl-2, PTEN, and AURKA Genes in OEO-Treated MCF-7 Cells

**Figure 2:**
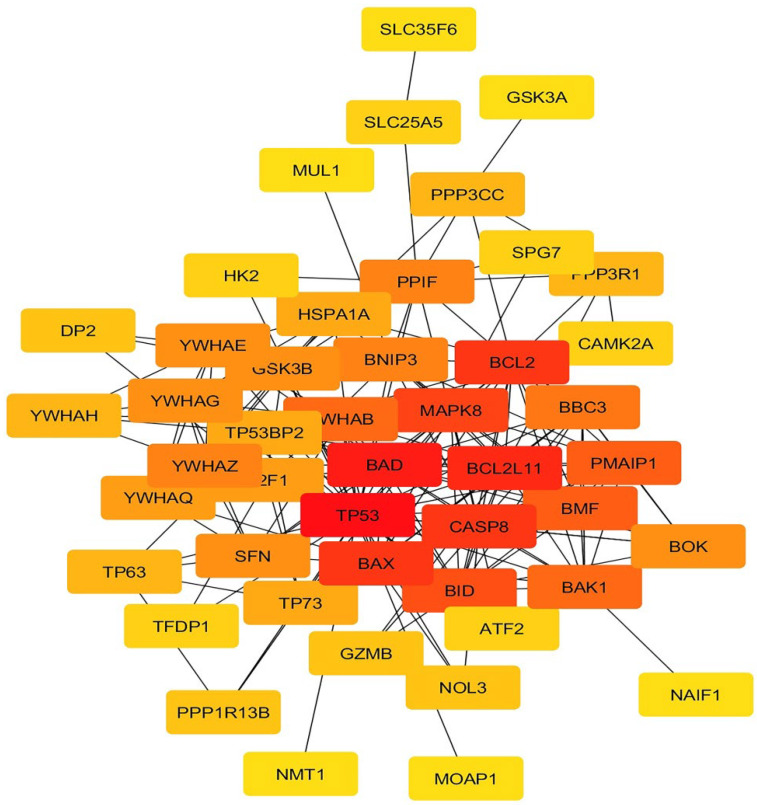
PPI (Protein–Protein Interaction) network and top hub genes. Hub genes were identified from all genes by using the degree analysis method. Genes’ ranking is based on the red spectrum. The higher gene rank is shown by red, while the color of lower ranking is yellow.

As can be seen in Figure 2 (colored nodes), hub genes were identified from all mitochondrial apoptotic genes in the network based on degree analysis. Two factors of *Bcl-2* and *BIM* (*Bcl2L11*) were selected for anti-apoptotic and pro-apoptotic genes, respectively. It was found that PTEN alterations and protein loss normally occur in breast cancer [24]. PTEN acts as a transcriptional repressor, inhibits cell survival signaling pathways (by AKT-pathway), and negatively regulates human breast carcinoma cell growth [25]. Furthermore, AURKA induces AKT and NF-KB pathways. Therefore, AURKA leads to tumor growth and proliferation through the mentioned pathways [26]. *PTEN* and *AURKA* are usually selected as repressor and proliferation genes, respectively.

According to the current study, molecular mechanisms were involved in the occurrence of these effects. The evaluations primarily relied on the ability of OEO to affect the expression levels of *Bcl-2*, *BIM*, *AURKA*, and *PTEN*. The expressions of *Bcl-2* and *AURKA* were reduced after a 48 h exposure to OEO (Figure 1E). By contrast, an increase in BIM and *PTEN* led to changes in *BIM/Bcl-2* and *PTEN/AURKA* and, consequently, caused apoptosis which inhibited tumor growth.

### 2.7. Relationship between Gene Expressions in OEO-Treated MCF-7 Cells

Various correlation analyses were carried out on *PTEN*, *BIM*, *AURKA*, and *Bcl-2*. According to Pearson’s correlation analysis, there was a positive correlation between the expression levels of *PTEN* and *BIM*, but a negative correlation between *PTEN* and *Bcl-2*. In addition, a positive correlation existed between *AURKA* and *Bcl-2*, but a negative correlation appeared between *AURKA* and *BIM*. There was a negative correlation between *BIM* and *Bcl-2* expressions (Table 3).

### 2.8. Analysis of Protein–Ligand Interaction of PTEN with Main Compounds of OEO

**Figure 3:**
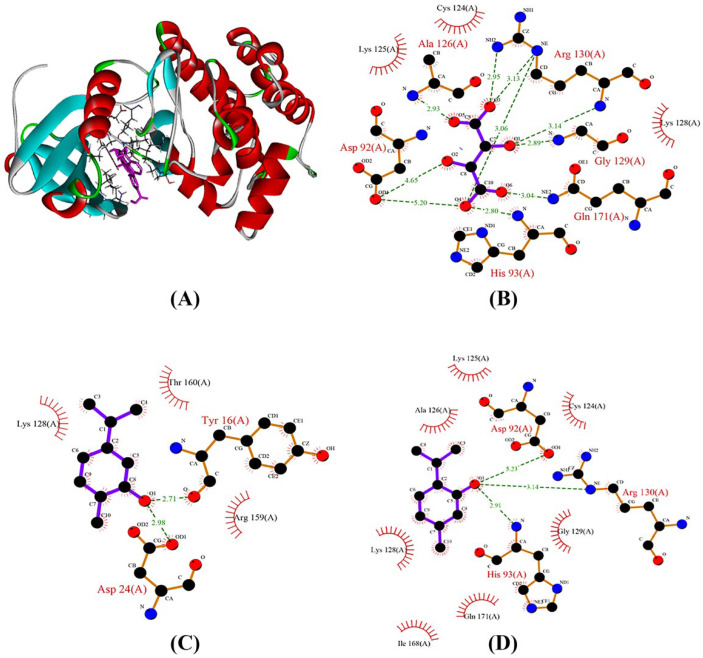
The 3D structure of PTEN bonds efficiently with the main compounds of OEO and TLA (**A**). Protein–ligand interaction profile of PTEN Tumor Suppressor Protein (PDB: 1D5R) with TLA (**B**), carvacrol (**C**), and thymol (**D**). Interactions are shown as colored lines: H-bond in target, green line–hydrophobic interaction, red colored lines.

The docking tests indicated that carvacrol and thymol were better ligands for the PTEN protein when compared to other compounds based on the docking score (Table 4). According to earlier reports, the amino acids Cys124, Arg130, His93, Gly127, Asp92, Gln171, Ala126, Lys125, and Lys128 determine the function of the PTEN protein [27]. Finding the potential binding mechanism of the major OEO compounds with the PTEN protein is the main goal of molecular docking. It was interesting to note that the creation of hydrogen bonds had a substantial impact on the interaction of the PTEN protein with thymol and carvacrol. Thymol demonstrated three hydrogen bond interactions with the amino acid residues Arg130, His93, and Asp92. Among these, Asp92 is a key residue of the PTEN catalytic pocket and creates a strong bond with the hydroxyl group of the thymol molecule. Additionally, carvacrol demonstrated two hydrogen bond interactions with the amino acid residues Tyr16 and Asp24 (Figure 3). Through hydrophobic interactions, both *p*-cymene and γ-terpinene docked. Overall, our findings suggest that thymol and carvacrol act as PTEN activators and may be useful in the treatment of breast cancer.

### 2.9. AURKA Interaction with Ligands during Molecular Docking

**Figure 4:**
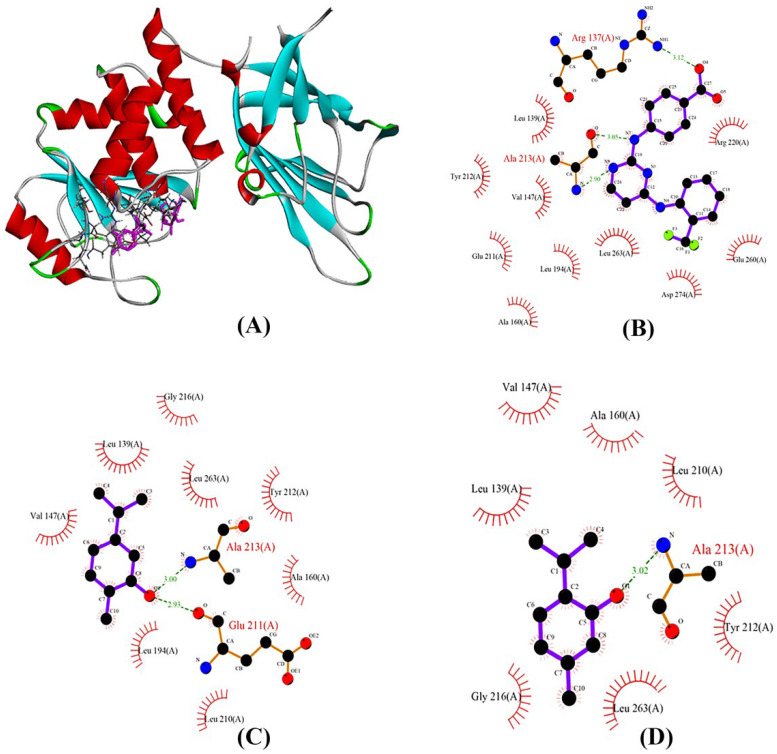
The interactions between AURKA (PDB ID: 3UOD) and OEO main compounds (**A**), OC3 (**B**), carvacrol (**C**), and thymol (**D**). Hydrogen bonds have been labeled using green dashed lines.

In this study, the docking approach was used for examining how the main compounds of OEO and OC3 interacted with AURKA. The results indicated that carvacrol and thymol are nearly docked at the OC3 binding site. As a result, H-bonds and hydrophobic interactions between either thymol or carvacrol and the AURKA active site were observed, resulting in acceptable ligand–receptor affinity (Figure 4). However, *p*-cymene and γ-terpinene docked virtually at an active location with a high docking score (Table 5) through a hydrophobic interaction. Figure 4 shows the protein–ligand complexes with hydrogen bond interactions. Carvacrol can develop H bonds with the Glu 211 and Ala 213 in the hinge region of the Aurora A (Figure 4). The hinge region of Aurora kinase A plays a crucial role in the formation of the catalytic active site and is located at residues 210–216 [28].

## 3. Discussion

Almost 74% of the new anticancer compounds are natural products or their derivatives [9]. The biological activity of EOs is associated with alteration of the cell membrane permeability and various intracellular targets. EOs may enhance intracellular ROS/RNS levels and trigger apoptosis in cancer cells [29]. Many different forms of cancer, including breast cancer, have been linked to the loss of PTEN function [30]. Some natural anticancer agents can stimulate or increase PTEN gene/protein expression/activity [31]. Aurora A kinase becomes an over-expressed gene in a variety of cancers, including solid tumors and leukemia [26]. In the current research, the OEO treatment increased PTEN expression and resulted in a significant reduction of AURKA, an established regulator of cell survival. OEO increased PTEN, thereby modulating the activity of the PI3K/Akt pathway downstream [32]. The potential anticancer activity of single OEO compounds against the MCF-7 cell line was not examined in the current study. Thus, at this stage, it was not possible to determine which compounds led to the effects seen herein.

OEO chemical analysis using GC/MS showed high percentages of thymol, carvacrol, γ-terpinene, and *p*-cymene. There was a significant decrease in γ-terpinene percentage, whereas thymol and carvacrol percentages increased through plant growth upon the full flowering stage. Thymol and carvacrol reached their highest percentages during the flowering stage [33]. The cytotoxic impacts of carvacrol on cancer cells have already been documented [34]. In addition, carvacrol was reported to decrease Bcl2/Bax and to induce apoptosis [35]. Thymol is endowed with anti-inflammatory, anticancer, antioxidant, and antimicrobial properties [36]. This compound exerts its anticancer effects through the suppression of cell growth, induction of apoptosis, production of intracellular ROS, depolarization of mitochondrial membrane potential, and activation of various pro-apoptotic mitochondrial proteins in human gastric AGS and breast cancer cells [37,38]. Thus, the antitumor, antioxidant, and antimicrobial activities of OEO [19,20,21] may be related to the presence of thymol and carvacrol. The induction of apoptosis and cell death are regarded as valuable effects of the OEO herein.

We described here the interaction of PTEN and AURKA with four major compounds of OEO. The structure of PTEN encompasses an N-terminal phosphatase domain (residue 7–185) and a C-terminal C2 domain (residue 186–351). The phosphatase domain includes the active site, which performs the protein’s enzymatic action, while the C2 domain binds to the phospholipid membrane [27,39,40]. Therefore, the C-terminus is involved in both protein stability and PTEN function. The OEO inhibited the breast cancer signaling pathway (PI3K/Akt) in tumor cells by increasing the expression level of the tumor-suppressor PTEN protein in MCF-7 cells. The docking showed that carvacrol and thymol were more effective than γ-terpinene and *p*-cymene in activating the PTEN. As a result, thymol may play a role in inducing apoptotic cell death via activating the PTEN protein.

The poor prognosis of cancer patients is related to the over-expression of AURKA. Thus, AURKA is regarded as a target for cancer treatment [41,42]. The development of AURKA inhibitors has become a major challenge in cancer treatment [43]. Noteworthy, the decrease in AURKA activity by OEO containing high percentages of thymol and carvacrol probably results in the blockage of the active site and reduction of enzyme activity. Thymol and carvacrol interacted with the main chain of AURKA by direct H-bonding, specifically the amino acid residues Glu211 and Ala213. These residues (Glu211 and Ala213) are considered hot spots because they significantly contribute to inhibitor binding interactions. Moreover, several of the structurally conserved AURKA proteins were Leu139, Glu211, and Ala213 [28,44]. Notably, the Ala213 residue is fundamental for the formation of essential H-bond interactions with ligands. These residues are needed for the catalytic activity of AURKA [28]. Thus, carvacrol and thymol are potent inhibitors of the AURKA protein in breast cancer. Studying the interactions of the main compounds of OEO can provide insights into their potential use in breast cancer treatment.

## 4. Materials and Methods

### 4.1. Preparation of O. decumbens Vent Essential Oil

Aerial parts of *O. decumbens* Vent. were collected at full flowering stage from Kazeroun, Fars province, south of Iran (29°35′41.0″ N latitude, 51°44′49.3″ E longitude). Herbarium specimens were recorded by Dr. Ahmad Reza Khosravi with number (55,075) and maintained at the Faculty of Science, Department of Biology at Shiraz University. After drying the plant materials in the shade, OEO was obtained through hydro-distillation using a Clevenger-type apparatus for approximately 4 h. After dehydration with anhydrous sodium sulfate, the OEO was stored in tightly closed dark vials at 4 °C. Finally, the OEO yield (%, *v*/*w*) was calculated as the weight of collected oil from dry material × 100. The extraction efficiency was 3%.

### 4.2. Identification of Oliveria decumbens Vent Essential Oil Components

By applying a flame ionization detector (FID) with the HP-5 capillary column (30 m × 0.32 mm i.d; film thickness 0.25 μm), the GC analysis was carried out on an Agilent 7890-A gas chromatograph (Agilent Technologies, Palo Alto, CA, USA). The injector and detector temperatures were 250 and 280 °C, respectively. Nitrogen, which was associated with a flow rate of 1 mL/min, was applied as the carrier gas. In addition, the range and rate of increase in oven temperature were 60–210 °C and 4 °C/min, respectively. Subsequently, the above-mentioned temperature increased to 240 °C (at a ratio of 20 °C/min). This temperature was isothermally maintained for 8.5 min. The split ratio was 1:50. The GC–MS analysis was carried out on an Agilent gas chromatograph, which was equipped with a fused silica capillary HP-5MS column (30 m × 0.25 mm i.d.; film thickness 0.25 μm) and the 5975-C mass spectrometer detector. Helium was applied as the carrier gas with an ionization voltage of 70 eV. Utilized ion source and interface temperatures were determined to be 230 and 280 °C, respectively, while the mass range was 45 to 550 amu. Conditions of the oven temperature program were similar to those of GC-FID. A homologous series of *n*-alkanes (C_8_–C_25_) was used to calculate the Retention Indices (RIs) of all components. Then, these values were compared with those reported in the literature [45]. In addition, related mass spectra were compared with those available in the Wiley/NBS data bank (Willey/ChemStation data system and NIST 08/National Institute of Standards and Technology). The peak area percentages were obtained by FID without applying correction factors.

### 4.3. DPPH Assay

The DPPH method was conducted to test the free radical-scavenging capability using the Blois technique [46]. The antioxidant activity of OEO was measured using the equation below.

% inhibition= {(Abscontrol − Abssample)}/(Abscontrol) × 100

All the examinations were triply performed with IC_50_ values, which were considered as mean values ± SD of triplicates. Compared to standard/commercial antioxidant (TBHQ), OEOs’ inhibitory concentration (IC_50_) had to inhibit 50% of DPPH radicals obtained from the standard curve.

### 4.4. Cell Culture

MCF-7 cells (National Cell Bank, Pasteur Institute of Iran, Teheran, Iran) were cultured in an RPMI 1640 medium (Bioidea Company-Iran, Teheran, Iran) with a pH value of 7.4. This medium was supplemented with 10% heat-inactivated fetal bovine serum (FBS) (Gibco-BRL, Baltimore, MD, USA), 1% penicillin (100 units/mL) (Biosera-Iran, Teheran, Iran), and streptomycin (100 μg/mL) (Biosera-Iran). The cells were also maintained in a humidified atmosphere of CO_2_ (5%) at 37 °C.

### 4.5. Cell Viability Test

To perform cell viability assays, cells were seeded in 96-well tissue culture plates at a density of 1.5 × 10^4^ cells/well in a 0.2 mL medium. The period of cell incubation was 24 h. The colorimetric MTT assay was applied to quantify cell viability. Applying the above-mentioned assay led to the measurement of dimethylthiazol diphenyl tetrazolium bromide reduction into formazan using mitochondrial enzyme succinate dehydrogenase. This reduction capacity represented the number of viable cells. After the 24 h period, the medium was discarded and 200 MTT solutions (Sigma-Aldrich, London, UK) (0.5 mg/mL final concentration) were added to the cells. Reading the absorbance was carried out at 570 nm after shaking through a micro-plate reader (Stat Fax 2100, Ramsey, MN, USA). As illustrated, IC_50_ values were calculated using GraphPad Prism 4 (GraphPad Software Inc., San Diego, CA, USA).

### 4.6. Acridine Orange/Ethidium Bromide Staining

Ethidium bromide (EB)/acridine orange (AO)-fluorescence labeling was used for measuring apoptosis and cell viability. The untreated and treated cells were harvested and centrifuged. Cold PBS was used for washing the cell pellets before adding the EB/AO solution (1:1, *v*/*v*) at a final concentration of 100 g/mL to the cell suspension. Finally, a fluorescence microscope was used for viewing the labeled cells (Olympus, BX51, Tokyo, Japan).

### 4.7. DNA Extraction

There are several properties associated with apoptosis, including cellular DNA fragmentation into low-molecular-weight oligomers. Grown cells transform into a confluence which is then incubated with various concentrations of doxorubicin (0.22 µg/mL) and OEO (39.54 µg/mL) for 48 h. Moreover, DNA was extracted using a modified CTAB method [47].

### 4.8. PPI Network Analysis and Hub Gene Identification

To construct the gene network and find intrinsic apoptosis hub genes, the PPI (Protein–Protein Interaction) analysis was carried out using STRING (http://string.embl.de/, accessed on 15 June 2021). Data visualization was made by Cytoscape (version 3.7.1; http://www.cytoscape.org/, accessed on 15 June 2021). The process of hub detection and subnetwork identification was carried out using CytoHubba. In addition, ‘Degree’ was employed as a topological analysis method, utilized in CytoHubba. Furthermore, the PPI network topology was analyzed using the Hubba plug-in via ‘Degree’.

### 4.9. RNA Extraction and cDNA Synthesis Genomics

The MCF-7 cell line was routinely maintained in the RPMI 1640 media and supplemented with 10% FBS. The total RNA was extracted through an RNX-Plus reagent kit (Cinnagen, Tehran, Iran) according to the manufacturer’s instructions. Then, the quantity and concentration were measured using a Nanodrop device (Thermo Fisher Scientific, Waltham, MA, USA), while the integrity and quantity of RNA were measured by the visual observation of 28S and 18S rRNA bands on a 1% agarose gel. A first-strand cDNA synthesis kit (Fermentas, Leon-Rot, Germany) was applied to synthesize cDNAs based on the manufacturer’s instructions. DNA-free total RNA (1 μg) was reverse-transcribed using oligo-dT primers (Fermentas, Leon-Rot, Germany), and finally, cDNA samples were stored at −20 °C for future use.

### 4.10. Primer Design and Quantitative Expression Examination

NCBI nucleotide database (NCBI GenBank) was used for retrieving gene sequences. Allele ID 7 and Vector NTI 11 software were employed for designing *PTEN*, *AURKA*, *BIM*, *Bcl-2*, and *β-actin* (internal control gene) primers (Table 6). The specifics of all primers were subsequently checked through BLAST. In addition, real-time quantitative analysis of gene expression was performed in a lineGeneK thermal cycler (Bioer, Hangzhou, China). Real-time PCR reactions were prepared in 20 μL total volume that contained 5 μL cDNA (diluted) of cells, 10 μL Master Mix (Real Q Plus 2-X Green Low ROX), and 0.7 μL of 100 pmol of each primer (forward and reverse). Cycling states were made from an initial denaturation step of 94 °C/10 min, which is considered a “hot start”, followed by 40 cycles with 94 °C/10 s at the mentioned annealing temperature for 40 s and 72 °C for 30 s. An extra melting curve analysis in the range of 50–95 °C was utilized in each qPCR reaction to confirm the specific amplification.

### 4.11. Binding Site and Docking

The specifics of molecular docking, structures of the PTEN tumor suppressor, and Aurora kinase A were obtained from a protein data bank (PDB) (https://www.rcsb.org/, accessed on 17 April 2021) with PDB ID 1D5R and 3UOD at high resolution. The three-dimensional structures of carvacrol (10364), thymol (6989), γ-terpinene (7461), and *p*-cymene (7463) were obtained from PubChem (https://pubchem.ncbi.nlm.nih.gov/, accessed on 17 April 2021). To confirm parameters for the docking experiments, the original ligand, OC3 (Trifluoromethyl) phenyl] amino} pyrimidin-2-Yl)amino]benzoic acid) in the 3uod.pdb structure, and TLA (L-Tartaric acid) in 1dr5.pdb were used. Using the USCF Chimera software, the protein and ligand were docked, and all potential conformations were retrieved using the default settings. Finally, all results were displayed with PYMOL software, and the docking scores were recorded as binding free energy (ΔG). Furthermore, we used LIGPLOT v.4.5.3 to plot the protein–ligand interactions, which can be downloaded from https://www.ebi.ac.uk/thornton-srv/software/LIGPLOT/, accessed on 30 June 2021. The active binding pockets of ID 1D5R and 3uod were predicted by CASTp (http://sts.bioe.uic.edu/castp/, accessed on 30 June 2021).

### 4.12. Statistical Analysis

The qPCR provided cycle threshold (CT) values which were used for computing the relative fold expression (2-ΔΔCT method). Statistical analysis was carried out via IBM SPSS Statistics software, version 16. In addition, an unpaired *t*-test was applied to perform statistical comparisons. *p*-values less than 0.05 were regarded as statistically significant.

## 5. Conclusions

In this study, OEO, characterized by thymol, carvacrol, *p*-cymene, and γ-terpinene, exhibited antiproliferative activity against breast cancer cells. In silico molecular docking simulation showed that thymol and carvacrol may establish proper bonds in the AURKA active site, and thus inhibit this enzyme while activating PTEN. Apoptotic death in OEO-treated MCF-7 cells was also detected using fluorescence microscopy and DNA fragmentation assay. The apoptotic mechanism and the occurrence of antiproliferation were confirmed by an increase in the Bim/Bcl2 ratio and the PTEN/AURKA ratio. Due to the fact that OEO showed antiproliferative activities against MCF-7 cell line, it is required that more investigations should be conducted to deepen the activity on other cancer cell lines and the mechanism of action of OEO’s main constituents. Overall, OEO can be regarded as a promising candidate for developing future anticancer drugs.

## Figures and Tables

**Table 1:** The chemical composition of *Oliveria decumbens* Vent. Essential oil.

No	Compounds	Class ^a^	RI ^b^	RI ^c^	Relative Percentage (%)
1	α-Thujene	MH	927	930	0.14 ± 0.05
2	α-Pinene	MH	934	939	0.18 ± 0.02
3	β-Pinene	MH	973	979	1.53 ± 0.08
4	Limonene	MH	1023	1029	1.00 ± 0.12
5	β–Phellandrene	MH	1021	1030	0.88 ± 0.10
6	*p*-Cymene	MH	1024	1024	19.95 ± 0.63
7	γ-Terpinene	MH	1050	1059	19.65 ± 0.30
8	*cis*-Limonene oxide	MO	1117	1132	1.09 ± 0.04
9	*trans*-Carveol	MO	1200	1215	0.46 ± 0.12
10	Thymol	MO	1271	1290	26.63 ± 1.42
11	Carvacrol	MO	1283	1298	24.12 ± 1.23
12	Eugenol	PP	1339	1356	0.14 ± 0.07
13	Spathulenol	SO	1566	1577	0.17 ± 0.11
14	Myristicin	PP	1494	1522	1.88 ± 0.24
Total	-		-		97.84

^b^ Retention indices experimentally determined on an HP-5MS column. ^c^ Retention index value taken from ADAMS library. ^a^ MH: Monoterpene Hydrocarbons; MO: Oxygenated Monoterpenes; PP: Phenylpropanoids; SO: Oxygenated sesquiterpenes.

**Table 2:** Growth inhibitory effects (IC_50_ values) of OEO and doxorubicin on the MCF-7 cell line after 24, 48, and 72 h of treatment.

Time (h)	OEO (µg/mL)	Doxorubicin (µg/mL)
24	84.07 * ± 6.66	-
48	39.54 * ± 2.35	0.215 * ± 0.028
72	33.32 * ± 1.14	0.111 * ± 0.036

Asterisks (*) indicate statistical significance caused by the essential oil (*p* < 0.01), as determined by the *t*-test. Data are the mean values of three replicates.

**Table 3:** Correlation between expressions of genes in MCF-7 cells in response to OEO.

		*Aurka*	*Bim*	*Bcl-2*
*PTEN*	Pearson’s r	−0.947 **	0.886 *	−0.967 **
	*p*-value	0.004	0.019	0.002
*Aurka*	Pearson’s r		−0.793	0.962 **
	*p*-value		0.060	0.002
*Bim*	Pearson’s r			−0.879 *
	*p*-value			0.021

** Correlation is significant at the 0.01 level (*p*-value). * Correlation is significant at the 0.05 level (*p*-value).

**Table 4:** Interaction of OEO compounds with PTEN.

Compound IDs	Docking Score	H-Bond Interactions	Hydrophobic Interaction
10364	−5.3	Tyr16, Asp24	Lys128, Arg159, Thr160
6989	−5.4	Asp92, His93, Arg130	Cys124, Lys125, Ala126, Lys128, Gly129, Ile168, Gln171
7461	−4.6	-	Tyr16, Asp24, Lys28, Gly127, Arg159, Thr160
7463	−4.7	-	Tyr16, Asp24, Lys28, Gly127, Arg159, Thr160

**Table 5:** Interactions between OEO compounds and AURKA.

Compound IDs	Docking Score	H-Bond Interactions	Hydrophobic Interaction
10364	−7.0	Glu211, Ala213	Leu139, Val147, Ala160, Leu194, Leu210, Tyr212, Gly216, Leu263
6989	−6.2	Ala213	Leu139, Val147, Ala160, Leu210, Tyr212, Gly216, Leu263
7461	−6.7	-	Leu139, Val147, Ala160, Leu194, Leu210, Tyr212, Ala213, Gly216, Leu263
7463	−6.8	-	Leu139, Val147, Ala160, Leu194, Leu210, Tyr212, Ala213, Gly216, Leu263

**Table 6:** Sequences of the primers used for RT-PCR amplification, product sizes, and Ta.

Gene	Primer Sequence 5′→3′	Ta (°C)	Product Length (bp)
*Bim*	F: CCACCAGCACCATAGAAGAAT	63	135
	R: TAAGGAGCAGGCACAGAGA		
*PTEN*	F: CAGTAGAGGAGCCGTCAA	58.5	108
	R: CAGAGTCAGTGGTGTCAGA		
*Aurka*	F: CATAGAGACATTAAGCCAGAGA	59	157
	R: GCATCCGACCTTCAATCA		
*Bcl-2*	F: AGTGATAATCAAGTCCTTT	60	155
	R: GGCAGTCCAGATGAACCG		
*β-actin*	F: GCCTTTGCCGATCCGC	65	160
	R: GCCGTAGCCGTTGTCG		

Ta, temperature annealing; F, Forward; R, Reverse.

## Data Availability

The datasets used and/or analyzed during the current study are available from the corresponding author upon reasonable request.

## Abbreviations

AKT	serine/threonine kinase
AURKA	Aurora kinase A
Bcl-2	b-lymphocytoma-2 gene
BIM	BCL2 like 11
CTAB	Cetrimonium bromide
DPPH	di(phenyl)-(2,4,6-trinitrophenyl) iminoazanium
GC/MS	Gas chromatography–mass spectrometry
MTT OEO	3-(4,5-dimethylthiazol-2-yl)-2,5-diphenyltetrazolium bromide *O. decumbens* essential oil
TBHQ	Tertiary butylhydroquinone
PiP3 Pi3K PPi PTEN	phosphatidylinositol (3,4,5)-triphoshpate phosphatidylinositol-3 kinase Protein Protein Interaction Phosphatase and tensin homolog

## References

[1] He Z., Chen Z., Tan M., Elingarami S., Liu Y., Li T., Deng Y., He N., Li S., Fu J. (2020). A Review on Methods for Diagnosis of Breast Cancer Cells and Tissues. Cell Prolif..

[2] Feng Y., Spezia M., Huang S., Yuan C., Zeng Z., Zhang L., Ji X., Liu W., Huang B., Luo W. (2018). Breast cancer development and progression: Risk factors, cancer stem cells, signaling pathways, genomics, and molecular pathogenesis. Genes Dis..

[3] Rodriguez S., Huynh-Do U. (2012). The Role of PTEN in Tumor Angiogenesis. J. Oncol..

[4] Álvarez-Garcia V., Tawil Y., Wise H.M., Leslie N.R. (2019). Mechanisms of PTEN Loss in Cancer: It’s All about Diversity. Semin. Cancer Biol..

[5] Bonneau D., Longy M. (2000). Mutations of the Human PTEN Gene. Hum. Mutat..

[6] Ton A.-T., Singh K., Morin H., Ban F., Leblanc E., Lee J., Lallous N., Cherkasov A. (2020). Dual-Inhibitors of N-Myc and AURKA as Potential Therapy for Neuroendocrine Prostate Cancer. Int. J. Mol. Sci..

[7] Tang A., Gao K., Chu L., Zhang R., Yang J., Zheng J. (2017). Aurora Kinases: Novel Therapy Targets in Cancers. Oncotarget.

[8] Curčić M.G., Stanković M.S., Mrkalić E.M., Matović Z.D., Banković D.D., Cvetković D.M., Dačić D.S., Marković S.D. (2012). Antiproliferative and Proapoptotic Activities of Methanolic Extracts from *Ligustrum vulgare* L. as an Individual Treatment and in Combination with Palladium Complex. Int. J. Mol. Sci..

[9] Abdullah A.-S.H., Mohammed A.S., Abdullah R., Mirghani M.E.S., Al-Qubaisi M. (2014). Cytotoxic Effects of *Mangifera Indica* L. Kernel Extract on Human Breast Cancer (MCF-7 and MDA-MB-231 Cell Lines) and Bioactive Constituents in the Crude Extract. BMC Complement. Altern. Med..

[10] Greenwell M., Rahman P.K.S.M. (2015). Medicinal Plants: Their Use in Anticancer Treatment. Int. J. Pharm. Sci. Res..

[11] Talib W.H., Daoud S., Mahmod A.I., Hamed R.A., Awajan D., Abuarab S.F., Odeh L.H., Khater S., Al Kury L.T. (2022). Plants as a source of anticancer agents: From bench to bedside. Molecules.

[12] Fitsiou E., Pappa A. (2019). Anticancer Activity of Essential Oils and Other Extracts from Aromatic Plants Grown in Greece. Antioxidants.

[13] Samadi N., Masoum S., Mehrara B., Hosseini H. (2015). Application of Linear Multivariate Calibration Techniques to Identify the Peaks Responsible for the Antioxidant Activity of *Satureja hortensis* L. and *Oliveria decumbens* Vent. Essential Oils by Gas Chromatography–Mass Spectrometry. J. Chromatogr. B.

[14] Li Y., Yeung C., Chiu L.C.M., Cen Y., Ooi V.E.C. (2009). Chemical Composition and Antiproliferative Activity of Essential Oil from the Leaves of a Medicinal Herb, *Schefflera heptaphylla*. Phytother. Res..

[15] Sibanda S., Chigwada G., Poole M., Gwebu E.T., Noletto J.A., Schmidt J.M., Rea A.I., Setzer W.N. (2004). Composition and Bioactivity of the Leaf Essential Oil of *Heteropyxis dehniae* from Zimbabwe. J. Ethnopharmacol..

[16] Yousefzadi M., Riahi-Madvar A., Hadian J., Rezaee F., Rafiee R., Biniaz M. (2014). Toxicity of Essential Oil of *Satureja khuzistanica*: In Vitro Cytotoxicity and Anti-Microbial Activity. J. Immunotoxicol..

[17] Sharifi-Rad J., Sharifi-Rad M., Hoseini-Alfatemi S.M., Iriti M., Sharifi-Rad M., Sharifi-Rad M. (2015). Composition, Cytotoxic and Antimicrobial Activities of *Satureja intermedia* CA Mey Essential Oil. Int. J. Mol. Sci..

[18] Karami A., Khoshbakht T., Esmaeili H., Maggi F. (2020). Essential Oil Chemical Variability in *Oliveria decumbens* (Apiaceae) from Different Regions of Iran and Its Relationship with Environmental Factors. Plants.

[19] Saidi M. (2014). Antioxidant Activities and Chemical Composition of Essential Oils from *Satureja khuzestanica*, *Oliveria decumbens* and *Thymus daenensis*. J. Essent. Oil Bear. Plants.

[20] Khajehie N., Golmakani M.-T., Eblaghi M., Eskandari M.H. (2017). Evaluating the Effects of Microwave-Assisted Hydrodistillation on Antifungal and Radical Scavenging Activities of *Oliveria decumbens* and *Chaerophyllum macropodum* Essential Oils. J. Food Prot..

[21] Khosravinezhad M., Talebi E., Shivakumar, Nemati Z., Nasrollahi I. (2017). Essential Oil Composition and Antimicrobial, Antioxidant Activities of *Oliveria decumbens* Vent. Int. J. Herb. Med..

[22] Ribble D., Goldstein N.B., Norris D.A., Shellman Y.G. (2005). A Simple Technique for Quantifying Apoptosis in 96-Well Plates. BMC Biotechnol..

[23] Hashemi M., Karami-Tehrani F., Ghavami S., Maddika S., Los M. (2005). Adenosine and Deoxyadenosine Induces Apoptosis in Oestrogen Receptor-Positive and -Negative Human Breast Cancer Cells via the Intrinsic Pathway. Cell Prolif..

[24] Depowski P.L., Rosenthal S.I., Ross J.S. (2001). Loss of Expression of the PTEN Gene Protein Product Is Associated with Poor Outcome in Breast Cancer. Mod. Pathol..

[25] Ghosh A.K., Grigorieva I., Steele R., Hoover R.G., Ray R.B. (1999). PTEN Transcriptionally Modulates C-Myc Gene Expression in Human Breast Carcinoma Cells and Is Involved in Cell Growth Regulation. Gene.

[26] Katsha A., Belkhiri A., Goff L., El-Rifai W. (2015). Aurora Kinase A in Gastrointestinal Cancers: Time to Target. Mol. Cancer.

[27] Lee J.-O., Yang H., Georgescu M.-M., Di Cristofano A., Maehama T., Shi Y., Dixon J.E., Pandolfi P., Pavletich N.P. (1999). Crystal Structure of the PTEN Tumor Suppressor: Implications for Its Phosphoinositide Phosphatase Activity and Membrane Association. Cell.

[28] Chinnasamy S., Selvaraj G., Kaushik A.C., Kaliamurthi S., Chandrabose S., Singh S.K., Thirugnanasambandam R., Gu K., Wei D.-Q. (2020). Molecular Docking and Molecular Dynamics Simulation Studies to Identify Potent AURKA Inhibitors: Assessing the Performance of Density Functional Theory, MM-GBSA and Mass Action Kinetics Calculations. J. Biomol. Struct. Dyn..

[29] Aggarwal V., Tuli H.S., Varol A., Thakral F., Yerer M.B., Sak K., Varol M., Jain A., Khan M.A., Sethi G. (2019). Role of Reactive Oxygen Species in Cancer Progression: Molecular Mechanisms and Recent Advancements. Biomolecules.

[30] Tan M.-H., Mester J.L., Ngeow J., Rybicki L.A., Orloff M.S., Eng C. (2012). Lifetime Cancer Risks in Individuals with Germline PTEN Mutations. Clin. Cancer Res..

[31] Kim D., Suh J., Surh Y., Na H. (2017). Regulation of the Tumor Suppressor PTEN by Natural Anticancer Compounds. Ann. N. Y. Acad. Sci..

[32] Li L.-L., Wei L., Zhang N., Wei W.-Y., Hu C., Deng W., Tang Q.-Z. (2020). Levosimendan Protects against Doxorubicin-Induced Cardiotoxicity by Regulating the PTEN/Akt Pathway. BioMed Res. Int..

[33] Esmaeili H., Karami A., Maggi F. (2018). Essential Oil Composition, Total Phenolic and Flavonoids Contents, and Antioxidant Activity of *Oliveria Decumbens* Vent. (Apiaceae) at Different Phenological Stages. J. Clean. Prod..

[34] Sampaio L.A., Pina L.T.S., Serafini M.R., dos Santos Tavares D., Guimaraes A.G. (2021). Antitumor Effects of Carvacrol and Thymol: A Systematic Review. Front. Pharmacol..

[35] Arunasree K.M. (2010). Anti-Proliferative Effects of Carvacrol on a Human Metastatic Breast Cancer Cell Line, MDA-MB 231. Phytomedicine.

[36] Kowalczyk A., Przychodna M., Sopata S., Bodalska A., Fecka I. (2020). Thymol and Thyme Essential Oil—New Insights into Selected Therapeutic Applications. Molecules.

[37] Jamali T., Kavoosi G., Jamali Y., Mortezazadeh S., Ardestani S.K. (2021). In-Vitro, in-Vivo, and in-Silico Assessment of Radical Scavenging and Cytotoxic Activities of *Oliveria decumbens* Essential Oil and Its Main Components. Sci. Rep..

[38] Kang S.-H., Kim Y.-S., Kim E.-K., Hwang J.-W., Jeong J.-H., Dong X., Lee J.-W., Moon S.-H., Jeon B.-T., Park P.-J. (2016). Anticancer Effect of Thymol on AGS Human Gastric Carcinoma Cells. J. Microbiol. Biotechnol..

[39] Mondal S.K., Sen M.K. (2020). Loss of Phosphatase Activity in PTEN (Phosphatase and Tensin Homolog Deleted on Chromosome Ten) Results in Endometrial Carcinoma in Humans: An in-Silico Study. Heliyon.

[40] Georgescu M.-M. (2010). PTEN Tumor Suppressor Network in PI3K-Akt Pathway Control. Genes Cancer.

[41] Boi D., Souvalidou F., Capelli D., Polverino F., Marini G., Montanari R., Pochetti G., Tramonti A., Contestabile R., Trisciuoglio D. (2021). PHA-680626 Is an Effective Inhibitor of the Interaction between Aurora-A and N-Myc. Int. J. Mol. Sci..

[42] Lee Y.H., Park J., Ahn S., Lee Y., Lee J., Shin S.Y., Koh D., Lim Y. (2019). Design, Synthesis, and Biological Evaluation of Polyphenols with 4, 6-Diphenylpyrimidin-2-Amine Derivatives for Inhibition of Aurora Kinase A. DARU J. Pharm. Sci..

[43] Wu F., Chu P., Chen G., Wang K., Hsu W., Ahmed A., Ma W., Cheng W., Wu Y., Yang J. (2022). Natural Anthraquinone Compound Emodin as a Novel Inhibitor of Aurora A Kinase: A Pilot Study. Chem. Biol. Drug Des..

[44] Martin M.P., Zhu J.-Y., Lawrence H.R., Pireddu R., Luo Y., Alam R., Ozcan S., Sebti S.M., Lawrence N.J., Schönbrunn E. (2012). A Novel Mechanism by Which Small Molecule Inhibitors Induce the DFG Flip in Aurora A. ACS Chem. Biol..

[45] Adams R.P. (2007). Identification of Essential Oil Components by Gas Chromatograpy/Mass Spectrometry.

[46] Molyneux P. (2004). The Use of the Stable Free Radical Diphenylpicrylhydrazyl (DPPH) for Estimating Antioxidant Activity. Songklanakarin J. Sci. Technol..

[47] Saadat Y.R., Saeidi N., Vahed S.Z., Barzegari A., Barar J. (2015). An Update to DNA Ladder Assay for Apoptosis Detection. BioImpacts BI.

